# Parsimony, homology and the continuity of stone tool use in the hominin clade

**DOI:** 10.1017/ext.2026.10018

**Published:** 2026-08-06

**Authors:** Tomos Proffitt, Jonathan Scott Reeves, Elisa Bandini

**Affiliations:** 1 Interdisciplinary Center for Archaeology and the Evolution of Human Behaviour (ICArEHB), University of Algarve, Portugal; 2 Department of History, Archaeology and Heritage Studies, Makerere University, Kampala

**Keywords:** last common ancestor, cladistics, parsimony, tool use, primates, early stone tools

## Abstract

Whether the last common ancestor (LCA) of *Homo* and *Pan* (*ca.* 6–7 Ma) used stone tools is a foundational question in palaeoanthropology. However, it is often treated as known rather than as a hypothesis requiring substantiation. Lacking fossil evidence for the LCA, this assumption is based on data from extant primates and the available hominin fossil record. Phylogenetic approaches invoking parsimony argue that the presence of stone tool use in both *Pan* and *Homo* reflects homology rather than convergent evolution. While methodologically legitimate, we argue that this framework has been applied without sufficient attention to the specific criteria for this to be true. For homology to hold, stone tool use must have been continuously expressed across generations of both lineages from the LCA to the present. We evaluate this claim against three lines of evidence: behavioural data from extant primates, showing that stone tool use is heterogeneous within *Pan*; the hominin fossil and archaeological record, which leaves a substantial evidential gap between the LCA and the earliest known documented technologies; and comparative data from phylogenetically distant tool using animals, which show that similar behaviours can arise independently under comparable ecological conditions. Together, these lines of evidence favour a model of multiple, context-dependent origins of stone tool use over unbroken inheritance from a tool using LCA.

## Impact statement

This study addresses an assumption about human origins: that our last common ancestor with chimpanzees and bonobos used stone tools. Rather than accepting this, the study reframes it as a testable hypothesis and shows that current evidence may not support a simple, continuous inheritance of stone tool use. By bringing together data from living primates, the archaeological record, and broader evolutionary comparisons, the paper suggests that tool use is flexible, context-dependent and capable of emerging independently in different species. This shift in perspective has important implications for how we study human evolution. It encourages researchers to move beyond simplified models and recognize the role of environmental conditions, learning and cultural transmission in shaping behaviour over time. It also highlights the limits of relying solely on genetic relatedness or parsimony when reconstructing the behaviour of extinct ancestors. More broadly, this work contributes to a growing understanding of technology as an adaptive behaviour that can arise multiple times under similar conditions, rather than as an inherited trait.

## Introduction

The extent to which our species uses tools is a defining characteristic of humanity. Its evolution is likely the result of a multi-million-year evolutionary relationship between tools, environments and our ancestors. As a result, when this relationship began is a fundamental question in paleoanthropology. Whilst the earliest physical evidence of tool use extends back to 3.3 Ma, there is a general consensus that hominin tool use likely emerged earlier (McPherron et al., 2010; Harmand et al., 2015; Lewis and Harmand, 2016; Thompson et al., 2019; Luncz et al., 2022). Furthermore, it is likely that some of the first tools used by hominins were made of organic materials predating the earliest lithic technologies (Whiten et al., 1999; Panger et al., 2003). However, evidence of potential organic tools is unlikely to be detectable because the archaeological record only preserves the most durable of materials (Barham et al., 2023). Thus, hominin tool use prior to the archaeological record is inferred from indirect lines of evidence.

Cladistic methods are used to infer that the last common ancestor (LCA) between *Pan* and *Homo* also used tools, by suggesting that tool use in both these genera is a homologous trait (McGrew, 1992, 2010; Panger et al., 2003; Carvalho et al., 2008; Rolian and Carvalho, 2017). This concept is predicated on the notion that it is more parsimonious to assume that the presence of tool use in *Pan* and *Homo* is due to shared ancestry than to explain how tool use arose convergently in both lineages (Panger et al., 2003; Carvalho et al., 2009). Since then, the notion that the LCA was, in fact, a tool user is seemingly commonplace in both primatological and archaeological studies and has even been used as a baseline assumption in guiding subsequent analyses (Marchant and McGrew, 2005; McGrew, 2010; Luncz et al., 2015; Rolian and Carvalho, 2017).

Although the cladistic approach constitutes a valid method for investigating the evolution of behaviour, determining the most parsimonious scenario requires more than comparing the behavioural repertoires of living humans and chimpanzees. With regards to the *Pan*-*Homo* LCA, the core principles and rules of these methods are often neglected, and the implications that can be drawn are sometimes overinflated in the literature (Sayers and Lovejoy, 2008; Bandini et al., 2021). Overlooking the often-stringent rules of these approaches may lead to conclusions on the abilities of the LCA that are not supported by the approaches themselves. In this paper, we outline the phylogenetic methods used to create hypotheses about the LCA’s material culture and argue that some of the assumptions on the tool use abilities of the LCA may oversimplify the story present in the great ape, fossil and archaeological record. In doing so, we identify points in the evolutionary history of both *Pan* and *Homo* that may confound the notion that tool use in the LCA is the most parsimonious conclusion. As such, ideas derived from cladistics related to hominin tool use behaviours should be treated as hypotheses that require further substantiation.

## Parsimony: Interpreting phylogenetic relationships based on cladistics

In the absence of comprehensive material and fossil record, methods borrowed from cladistics have been used to hypothesize whether the LCA between *Pan* and *Homo* was a tool user (Duda and Zrzavý, 2013). Cladistics refers to a biological classification in which organisms are grouped by how closely they are related in time and by any existing shared traits. Traditionally, the method provides a way to determine if the presence of specific traits is the result of shared ancestry (homology) or independent evolution (homoplasy). By doing so, the technique allows us to establish evolutionary relationships among taxa and generate hypotheses about how traits evolved (Sober, 1991). In any given cladogram, there are likely several competing hypotheses regarding the origin of specific traits, and parsimony is one of the primary tools used to differentiate between them.

Parsimony is a theoretical principle that, among competing hypotheses, simpler explanations should be preferred over more complex ones (Wiley and Lieberman, 2011) (see also ‘Ockham’s razor’). Its value lies in limiting post hoc “just-so” narratives to justify conclusions by requiring that developmental reconstructions minimize the number of evolutionary steps needed to explain the origin of specific traits. When comparing tool use in both *Pan* and *Homo*, parsimony is often used to argue that it is simpler to conclude that tool use originated in a common ancestor rather than it is to explain how tool use may have emerged independently in the two closely-related taxa (McGrew, 1992), or indeed appear and become extinct multiple times in each species’ evolutionary history.

Discerning which scenario is most parsimonious is relatively straightforward for anatomical morphological traits, but its application to behavioural traits raises additional issues. Unlike morphology, whose transmission mechanisms and comparatively well understood, the transmission of behavioural traits is more complex (Ammar et al., 2023). The capacity for specific behaviour does not guarantee its habitual expression, and its absence in the record does not imply lack of capacity. Behavioural expression depends on multiple factors, including local ecology, individual and social learning, demographic structure and resource distributions. These factors are important because parsimony-based inferences of homology assume not only phylogenetic relatedness but also trait stability over time (Sober, 1991).

Although tool use may appear consistent with arising from the LCA of *Pan* and *Homo*, the homology claim calls for a stronger requirement than is often made explicit. If tool use in *Pan* and *Homo* is homologous, then tool use (or at least the assumed shared tool use trait) must have been conserved across the ancestral lineages from the LCA to each crown taxon. In other words, homology is not supported by the presence of tool use in the (current) endpoints of a lineage alone.

Therefore, for the *Pan-Homo* LCA question, the central issue is not whether parsimony is a legitimate principle, but whether the assumptions required to apply it to tool use as a behavioural trait are satisfied by the available evidence. While organic tool use is widespread among extant primates, its perishable nature renders it archaeologically invisible in Plio-Pleistocene contexts (Pascual-Garrido et al., 2024). By contrast, stone tool use, specifically the production of knapped lithics or percussive artefacts, provides a durable and testable record for examining the emergence and early record of technology. Although formal phylogenetic character mapping may offer a potential avenue for testing such hypotheses, its application in this context is constrained by the uncertain attribution of lithic behaviours to specific hominin taxa and by preservation and sampling biases that complicate reliable coding of both presence and absence.

## Evidence from non-human animals

### Evidence from Pan

Stone tool use in extant primates provides a critical comparative basis for evaluating whether this behaviour in *Pan* and *Homo* is plausibly homologous because it allows for the assessment of behavioural variation both within *Pan* and across other stone tool using primates. If stone tool use were an evolutionarily stable, widely shared behaviour within a given primate taxon, one would expect its expression to be broadly consistent among the extant members of that taxon.

Inferences regarding the LCA’s ability to use stone tools are often grounded in primatological data from chimpanzees and are primarily restricted to a well-studied subspecies of chimpanzees: *Pan troglodytes troglodytes* (Gruber and Clay, 2016). This is despite there being four (currently) known subspecies of wild chimpanzees that correspond to different regions in Africa ( *P. troglodytes troglodytes* in central Africa, *Pan troglodytes* . *schweinfurthii* in eastern Africa, *Pan troglodytes* *verus* in western Africa and *P. troglodytes* *eliotti* in Nigeria and Cameroon (Gruber and Clay, 2016). All of these subspecies vary in their tool use repertoires (Whiten et al., 1999; Kalan et al., 2020), and stone tool use is seemingly limited to the Western chimpanzees (although see the potential first report of nut-cracking with stone tools in Cameroon; Morgan and Abwe, 2006). These observed patterns of behavioural variation across chimpanzee subspecies suggest that stone tool use repertoires may have evolved locally within populations, even at a relatively fast pace, thus rendering the idea of one fixed chimpanzee model unrealistic (Langergraber et al., 2011; Rolian and Carvalho, 2017). With regards to evidence of stone tool use in the past, the primate archaeological record of the Taï Forest chimpanzees (in Cote d’Ivoire) currently provides the most reliable direct evidence for the antiquity of stone tool use in this subspecies (Mercader et al., 2002, 2007; Proffitt et al., 2018a). Excavations of the nut-cracking site of Noulo indicate that cracking encased nuts with stone tools has been practiced in the Taï Forest for around 4,300 years (Mercader et al., 2007). Furthermore, more recent excavations indicate that the neighbouring Djourotou chimpanzee group in the south of the Taï Forest has been using stone tools for at least 719 years (Proffitt et al., 2024). Beyond this, evidence for tool use in past members of *Pan* is scant. Primate archaeologists are yet to discover a *Pan* tool using site from the deep past. Moreover, the chimpanzee fossil record is only represented by a handful of fossils (McBrearty and Jablonski, 2005).

As result, studies on tool use in the *Pan* lineage focus on shared behaviours of the living subspecies. In this vein, some have questioned whether the considerable genetic differences within their own subspecies make chimpanzees reliable models for the LCA (Sayers and Lovejoy, 2008). Bonobos, equally related to humans, are rarely used likely due to their limited tool use (especially stone tool use) in the wild and captivity (Gruber and Clay, 2016; Bandini et al., 2026). Prioritizing chimpanzees as models for LCA tool use risks circular reasoning. Selecting a tool using taxon makes a tool using ancestor more likely. If bonobos were the only reference, such assumptions would be far weaker.

Gruber and Clay (2016) suggest a triangulation approach, incorporating bonobos, chimpanzees and modern humans, inferring that traits present in all three were likely present in the LCA. While more comprehensive, this approach is constrained by the 6–7 million years of independent evolution separating extant apes from the LCA (Rolian and Carvalho, 2017). This approach may still, however, suggest LCA tool use since chimpanzees and humans frequently use tools and bonobos show limited capacity (in the wild). However, capacity does not guarantee expression, and low frequency or absence of tool use may reflect ecological and/or social conditions rather than absence of capacity, as seems to be the case with extant bonobos (Koops et al., 2014; Gruber and Clay, 2016; Bandini et al., 2026). Moreover, behavioural variability within *Pan* complicates treating chimpanzee tool use as a single behaviour that maps cleanly onto the *Pan* – *Homo* LCA.

### Evidence from other animals

Comparative data from other animals also demonstrates that similar stone tool use behaviours can arise in lineages that are not closely related phylogenetically with either *Pan* or *Homo* and are more likely consistent with convergent evolution under different ecological conditions. One example comes from wild long-tailed macaques ( *Macaca fascicularis aurea* ) in Southeast Asia. Long-tailed macaques show a range of stone-tool-mediated percussive foraging behaviours, including shoreline-focused exploitation of gastropods, oysters and crabs, and in some cases inland nut-cracking using stone tools and anvils (Carpenter, 1887; Malaivijitnond et al., 2007; Gumert et al., 2019). The presence of behaviour-specific wear patterns on these macaques’ tools further supports the inference that broadly comparable percussive behaviours can yield archaeologically recognisable traces (Haslam et al., 2013, 2016; Falótico et al., 2017; Luncz et al., 2017; Proffitt et al., 2018b, 2021). Reports that other subspecies of long-tailed macaques ( *Macaca fascicularis fascicularis* ), not previously observed using tools, can shift to tool-related foraging in response to abrupt changes in resource availability (for example, during the COVID-19 pandemic, when reduced tourist provisioning was followed by increased stone-tool foraging), underscore the potential “fallback” role of tool use under certain ecological constraints (Muhammad et al., 2024).

However, habitual tool use continues to be heterogeneous within long-tailed macaques, suggesting a potential genetic link to behavioural expression (Bandini and Tennie, 2018; Gumert et al., 2019), although see again the new report of tool use in the subspecies that does not regularly use tools ( *M. fascicularis fascicularis* ; Muhammad et al., 2024).

Several groups of robust capuchin monkeys (*Sapajus* spp.) provide an additional comparative case of habitual stone tool use outside of chimpanzees. Multiple populations of capuchins (*e.g.*, *Sapajus libidinosus* , *Sapajus xanthosternos* , *Sapajus flavius* , *Cebus capucinus imitator* *)* use stone tools as part of daily foraging strategies, with percussive behaviours forming a recurring theme across sites (Fragaszy et al., 2004; Falótico and Ottoni, 2016; Barrett et al., 2018; Lima et al., 2024; Proffitt et al., 2025). In Brazil’s Serra da Capivara National Park (SCNP), capuchins habitually use stone hammers (with stone and occasionally wooden anvils) to crack nuts and process other foods (Falótico and Ottoni, 2016). Primate archaeological excavations indicate the presence of stone tool use at some localities extending to at least ~3,000 years ago (Falótico et al., 2019), suggesting long-term persistence alongside site-specific patterning shaped by raw material availability and food properties. These capuchins also use stones for digging and other subsistence and social behaviours (e.g., social display; Falótico and Ottoni, 2016), and additional tool-using populations have been identified elsewhere in central Brazil (e.g., Fazenda Matos and Ubajara National Park (Falótico et al., 2024; Proffitt et al., 2025). At Fazenda Boa Vista (FBV), Piaui (Brazil), the capuchins use stone hammers and large stone and wooden anvils to crack a variety of nut species (Fragaszy et al., 2004). It has been suggested that this stone tool use is multifaceted, with preferential selection of hammerstone size based on the hardness of the nut to be cracked (Visalberghi et al., 2007, 2009; Fragaszy et al., 2010, 2013). Furthermore, it has been shown that nut position on the anvil is highly controlled to ensure increased efficiency when cracking (Liu et al., 2011). At both SCNP and FBV, the local ecological setting is that of open dry woodland, known as Caatinga, where hard encased nuts and fruit make up a substantial portion of the capuchins’ diet.

Recently, observations of white-faced capuchins ( *C. capucinus imitator* ) in the islands of Coiba National Park in Panama using stone tools have also been reported (Barrett et al., 2018). These capuchins occupy a substantially different ecological niche from the capuchins in Brazil. These white-faced capuchins inhabit coastal environments and use stone hammers and anvils to crack open various nuts, gastropods and crabs, reminiscent of the stone tool use behaviours of long-tailed macaques in Southern Thailand (Barrett et al., 2018). A study of this behaviour found that stone tool use is practiced primarily by males, and that different levels of stone tool use is observed across the islands, despite the presence of adequate stone and encased food resources throughout (Goldsborough et al., 2024). Examples of habitual stone tool use are also found outside of primates. For example, one subspecies of otters ( *Enhydra lutris* ) has been observed to use stones to crack open encased food sources, such as shellfish and mussels (Fujii et al., 2015), and several bird species use stones as projectile weapons to crack open eggs (Marks and Hall, 1992; Debus, 2021; Bandini et al., 2022).

The existence of such a wide array of stone tool use types and extents across phylogenetically distant animals over such great geographic scales and niches indicates that similar behaviours do not necessarily reflect shared ancestry but may instead reflect repeated innovation as a consequence of existing abilities, external ecological pressures and/or social conditions.

These findings epitomize the fact that the ability to use tools can vary dramatically in animals, even between subspecies. In fact, the taxa that use stone tools the most in the wild (*Pan, Macaca, Sapajus* and *Cebus*) are not all sister taxa (Rolian and Carvalho, 2017). Therefore, whilst extant primates can provide insight into certain aspects of LCA behaviour, it is clear that the biological and behavioural mechanisms underlying stone tool use are more complex than simply phylogenetic distance. Taken together, extant animal evidence indicates that stone tool use is not a stable, uniformly expressed character within closely related taxa, and that convergent stone tool traditions can arise in distantly related lineages. This does not falsify the possibility of tool use in the *Pan* – *Homo* LCA, but it does weaken the common inference that homology is the default parsimonious explanation and motivates closer scrutiny of the fossil and archaeological records for continuity (or discontinuity) through time.

Some of these issues would be resolved by examining the antiquity of stone tool use in the *Pan* lineage. Whilst studies over the last two decades show that primates produce their own long-term archaeological record, its time-depth, for now, is superficial when compared to the temporal scale of the hominin record (Mercader et al., 2007; Proffitt et al., 2024). Furthermore, only one fossil chimpanzee has been discovered to date (McBrearty and Jablonski, 2005). Therefore, until we have fossil evidence of the LCA, it is difficult to assess whether chimpanzees truly are the most appropriate models. Other primate species, that are more phylogenetically distant but have more stone tool use behaviours within their repertoires (such as capuchins or macaques; Bandini et al., 2022), may actually prove to be more useful models for assessing under which conditions this type of tool use emerges, and whether the LCA met these conditions (McGrew et al., 2019). However, anatomical capacity represents a fundamental constraint that transcends behavioural observation. Stone tool use, particularly percussion and precision manipulation, demands specific finger proportions, thumb opposition and palm morphology that vary significantly across extant tool-using primates and early hominins (Dunmore et al., 2023).

## Evidence from the fossil and archaeological record

There is not a single community of living humans that does not use tools. There is also good evidence that our ancestors used tools over the course of the last 3.3 million years (Key and Williams, 2026). This archaeological record offers critical insights into how stone tool use persisted and transformed within the hominin lineage, as well as how it might trace back to the *Pan* – *Homo* LCA. However, the strength of inference varies substantially across hominin taxa and across time.

### Homo

Evidence for continuous tool use within the hominin lineage is clearest within the genus *Homo*, where stone tools and tool-related activities are supported by both a fossil and an extensive archaeological record over the past *circa* 2.6–2.9 million years (Braun et al., 2019, 2025; Plummer et al., 2023). There is no doubt that anatomically modern humans and their European counterparts, Neanderthals, were habitual tool users (Ambrose, 2001; D’Errico, 2003; Shea, 2017). After around 1.8 Ma, there is clear evidence of lithic assemblages spanning three continents and commonly associated with a diversity of *Homo* species, including *H.* *heidelbergensis* *, H.* *helmei* *, H.* *rhodesiensis* *, H.* *ergaster* *, H.* *erectus* and *H.* *antecessor* (Gabunia et al., 2001; Dennell and Roebroeks, 2005; Dennell and Petraglia, 2012; Shea, 2013). Whilst it is often difficult to directly attribute individual archaeological assemblages to specific members of the *Homo* lineage, the density and persistence of the record during the time range of *Homo* make habitual stone tool use very likely. Prior to 1.6 million years ago, the association of early *Homo and Homo* *ergaster* with Oldowan contexts further supports the continuous use of tools within the genus *Homo* (Leakey, 1971; Semaw et al., 2020).

### Paranthropus

*Paranthropus* (2.8–1.0 Ma) provides a potentially important test case, as it overlaps temporally and geographically with early *Homo* in East Africa. Multiple lines of evidence now suggest that *Homo* and *Paranthropus* may have been on different evolutionary trajectories (O’Brien et al., 2023). While tool use is strongly implicated for early *Homo*, whether *Paranthropus* made or used tools remains unresolved. *Paranthropus* fossils, however, are more often found in association with stone artefacts than members of the genus *Homo* (Leakey, 1971). For example, *Paranthropus robustus* is often found in association with artefacts in South Africa (Wood and Boyle, 2016). In East Africa, the *Paranthropus boisei* holotype (OH 5) was recovered in direct association with stone tools at FLK Zinj (Leakey, 1971), and *P.boisei* remains were also recovered from the FxJj 20 site complex in East Turkana (Isaac and Isaac, 1997). On the basis of the OH 5 association, some researchers proposed that *P. boisei* made and used tools, but this attribution was later reconsidered after the discovery of *Homo habilis* at the nearby site of FLK North (Leakey, 1959; Leakey et al., 1964).

Differences in dental morphology and isotopic signature between the two genera have been used to suggest that *Homo* was more of a generalist and that it used tools to expand its dietary niche (Schick and Toth, 1994; Wood and Constantino, 2007). In contrast, *Paranthropus’* large teeth and masticatory apparatus, as well as isotopic signatures, have been taken to suggest a heavier reliance on specialized adaptations, potentially reducing its dependence on tools, or not requiring tool use at all (Cerling, 1992; Schick and Toth, 1994). More broadly, association-based attribution is inferentially fragile: the same archaeological contexts often contain multiple hominin taxa, and when attribution is uncertain, it commonly defaults to *Homo*, even when *Paranthropus* fossils are present. This history matters because interpretations at Olduvai shifted as new taxa were discovered, illustrating how quickly “who made the tools” can change when additional fossils appear. Recent discoveries have reopened the question of *Paranthropus* technological behaviour. Hand and wrist remains attributed to *P. boisei* at around 1.5 Ma have been interpreted as compatible with human-like grips for pounding/cutting (Mongle et al., 2025). In addition, Oldowan assemblages from at Nyayanga on the Homa Peninsula (Kenya), dated to around 2.8 Ma, were found in close association with *Paranthropus* fossils and cut-marked fauna (Plummer et al., 2023). Further afield, *Paranthropus* has been reported at Mille-Logya (Afar, Ethiopia), dated to between 2.5 and 2.9 Ma (Alemseged et al., 2026), extending both its geographic range and its chronological overlap with early Oldowan occurrences.

Taken together, the *Paranthropus* evidence is best treated as *equivocal.* There are plausible cases for stone tool use and/or tool involvement at certain sites, but the current record does not support a strong claim of uniform, habitual lithic production across the clade. Several non-exclusive scenarios remain compatible with the data: (i) *Paranthropus* did not use stone tools and any association with archaeological remains is fortuitous; (ii) *Paranthropus* used stone tools opportunistically but was not a consistent stone tool maker; (iii) stone tool use was geographically restricted; (iv) stone tool use occurred but is archaeologically indistinguishable from contemporaneous Oldowan assemblages attributed to early *Homo*; or (v) stone tool use capacity existed but its expression was environmentally or regionally constrained and/or episodic. These possibilities raise a broader evolutionary question. Given *Paranthropus’* anatomical capacity and its growing chronological and geographic overlap with Oldowan contexts, is it more parsimonious to infer that lithic production originated outside the *Paranthropus* lineage, or that some degree of tool competence existed in *Paranthropus* but was expressed only episodically or in restricted regions? Resolving this requires evidence that goes beyond co-occurrence, ideally linking taxa to specific behavioural signatures through tighter stratigraphic control, more diagnostic activity traces, and clearer criteria for attribution.

### Beyond Homo and Paranthropus

Outside of *Homo* and *Paranthropus*, assessing tool use becomes increasingly difficult due to the limited fossil and archaeological record. Some have suggested *Australopithecus garhi* as an author of the 2.6 mya Oldowan assemblages from Gona (Schick and Toth, 2006) However, this suggestion has been weakened by the discovery of *Homo* from Ledi-Geraru at 2.8 mya (Villmoare et al., 2015). 3.3-million-year-old cut-marked bones from Dikika, Ethiopia also indicate that *A. afarensis* used tools. The similarly aged site of Lomekwi 3 in Kenya also suggests that *Kenyanthropus* could have been a tool user. The frequency to which they used tools is, however, unknown. In addition, these sites remain the focus of a fair amount of debate regarding their age and context (Domínguez-Rodrigo et al., 2012; Domínguez-Rodrigo and Alcalá, 2019; Archer et al., 2020).

As a result, discussions of tool use in hominins prior to 3.3 Ma are inferred from anatomical traits. Experimental studies suggest that producing Oldowan and Acheulean tools relies on a range of forceful precision grips (Marzke and Shackley, 1986; Marzke and Marzke, 1987; Marzke, 2013; Key et al., 2018). These grips are often associated with distinct anatomical traits of the hand, specifically the presence of a long thumb and apical tuffs. While these anatomical features are present in the *Homo* lineage, their presence in earlier members of the hominin clade may indicate a capacity for tool use even though this does not, by itself, demonstrate that such capacities were always behaviorally expressed (Kivell et al., 2023).

The fossil evidence supports the notion that *A. afarensis* and *A. africanus* may have possessed the anatomical ability to use the precision pad to side grip required for tool use, albeit without the ability to exert the amount of force seen in later *Homo* species (Ricklan, 1987; Kivell, 2015; Skinner et al., 2015). Furthermore, recent studies of trabecular bone structure in these species also suggest that tool use may have been practiced (Skinner et al., 2015). During the Early Pliocene, it has been shown that the metacarpal length of *Ardipithecus ramidus* is similar to that of later hominins, including *Australopithecus* and early *Homo* (Lovejoy et al., 2009). However, the proportions of its thumb to finger length place it somewhere between chimpanzees and gorillas, making the required precision grips for tool use difficult to undertake (Skinner et al., 2015). This does not preclude *A. ramidus* as a tool user; however, the likely inability to exert forceful precision grips used for stone tool production would limit the range of motion required to make stone tools. However, identification of stone tool use in extant wild capuchins and long-tailed macaques may point to alternative models of stone tool use, suggesting that such hand morphologies may be adapted to it. It has further been suggested that the broad distal phalanx of *Orrorin tugensis*, dated to around 6 Ma, may have also been conducive to tool use, or at least the ability to finely manipulate objects (Almécija et al., 2010).

## Discussion

Collectively, these data suggest that evidence for habitual stone tool use in pre-*Homo* taxa remains somewhat uncertain. The Plio-Pleistocene archaeological record is sparse, contested, or temporally overlapping with early *Homo.* Hominin anatomy can demonstrate capacity, but not direct evidence of behaviour, and therefore provides limited grounds for inferring habitual tool use. Therefore, it is difficult to confidently support a continuous lineage of stone tool use spanning the Miocene, Pliocene and Pleistocene as might be expected if such behaviour were conserved from the *Pan* – *Homo* LCA. For the parsimony hypothesis to hold, one must accept an absence of direct behavioural evidence over several million years, an assumption that considerably weakens the explanatory power of parsimony in this case.

The contrast between chimpanzee and bonobo tool use provides a useful analogue to the *Paranthropus* dilemma. Is it more parsimonious to posit that the LCA habitually used tools, with bonobos subsequently losing this capacity or motivation to do so, or that chimpanzees developed their habitual tool use behaviours independently? Without fossil evidence from *Pan* or the LCA and given the fragmentary nature of the early hominin record, these questions are unlikely to be resolved without new data.

The greatest challenge to parsimony-based arguments arises from the substantial temporal gap between the estimated age of the *Pan*–*Homo* LCA (*ca.* 6–7 Ma) and the earliest known knapped stone tools from Lomekwi 3 (*ca.* 3.3 Ma; attributed to *K. platyops*). This predates definitive *Homo* fossils by roughly 300 ka, while subsequent Oldowan assemblages (2.9–1.5 Ma) mark the emergence of small flake-based industries that persisted for over a million years. This 3–4 Ma hiatus encompasses a succession of late Miocene and early Pliocene hominins, none yet associated with direct evidence of stone tool use. Although hand morphologies in some of these taxa appear compatible with precision and power grips, such inferences indicate potential rather than practice and cannot demonstrate tool production as opposed to opportunistic use. If stone tool use were truly homologous and inherited from the LCA, it is difficult to explain its apparent absence over this vast timespan. Several alternatives are at least as parsimonious: (1) stone tool use emerged and went extinct multiple times within the hominin clade in response to ecological pressures; (2) it was lost and later re-evolved in specific taxa; (3) early hominins had the capacity but not the ecological, motivational, or cultural impetus to use tools; or (4) tools existed but remain archaeologically invisible due to preservation or recognition biases.

*Paranthropus boisei* is also an interesting case for parsimony. While there has been a recent resurgence in suggestions that *P. boisei* was a tool user, some maintain that it was not a tool user given its highly specialised diet. If the latter is the case, then the species poses issues for narratives arguing that the *Pan-Homo* LCA was a tool user due to homologous tool use in living *Pan* and *Homo.* If *P. boisei* did not use tools then, under this argumentation, it would imply that its ancestors were once tool users and then stopped. Then one would have to explain the circumstances under which *Paranthropus* lost the ability to use tools. One might question how parsimonious this scenario is, given how highly adaptive tools are assumed to be to the hominin clade. The key issue, then, is whether the current pattern reflects the absence of lithic technology in the *Paranthropus* lineage, or a behavioural capacity that was real but variable, and therefore difficult to detect archaeologically.

Given the vast range of phylogenetically disparate primates, and other animals for that matter, that use tools, it may be more parsimonious to suggest that ancestors of *Pan* and *Homo* converged on tool use at multiple points throughout evolutionary history.

Reframing early stone tool use as the outcome of multiple, context-dependent innovations provides a more flexible and empirically consistent model. Rather than an inevitable inheritance from an ape-like ancestor, tool use can be seen as a recurrent adaptive response to particular ecological and cultural circumstances. This perspective accommodates the long temporal gap preceding the earliest tools, explains the patchy distribution of tool use across primates and other animals and aligns with increasing recognition of early hominin diversity as a process of adaptive radiation involving multiple behavioural and technological pathways.

In addition, the evidence from primates and the hominin record shows that tool use is ecologically contingent and evolutionarily variable. Closely related species and even neighbouring populations can differ substantially in the presence, frequency and complexity of tool use, despite shared ancestry. In hominins, stone tool evidence is similarly patchy and discontinuous, even where anatomy permits fine manipulation. While these patterns are better explained by independent origins and context-dependent expressions than a single origin with repeated loss or long-term non-expression, this interpretation remains contingent on the available evidence. The discontinuous and relatively late-emerging lithic record does not support the idea of unbroken behavioural continuity from the LCA. Instead, archaeological evidence appears in discrete temporal and taxonomic clusters separated by long intervals with no recognizable tools.

## Conclusion and future directions

Treating *Pan* – *Homo* LCA stone tool use as an untested baseline assumption is oversimplistic and should be treated as a testable hypothesis. Claims of homology are often accepted as most parsimonious without considering implications for extinct ancestors or the growing evidence that tool use is more complex than *Pan* and *Homo* comparisons suggest. Greater behavioural and taxonomic diversity within *Pan* further complicates such inferences, as does the hominin record. While cladistics remain valuable, behavioural hypotheses must be empirically grounded.

Organic tool use may plausibly characterize the LCA but is largely archaeologically invisible for most of the Plio-Pleistocene. In contrast, habitual stone tool production, a complex technological behaviour, restricted to specific contexts and taxa and seemingly absent in multiple morphologically capable lineages, lacks the continuity expected under strict homology from the LCA. The temporal gap between LCA (6–7 Ma) and the earliest stone tools (3.3 Ma), the heterogeneous distribution of lithic evidence and the recent emergence of non-human primate stone tool traditions all argue against shared ancestral presence.

Future research should critically reassess how parsimony is invoked in behavioural reconstructions, refine the chronology of early tool use in hominins and primates and investigate the ecological and cultural drivers of stone tool use emergence. Finally, models of hominin evolution should allow the possibility that early stone tool use emerges through independent innovations in different lineages.

Overall, phylogenetic methods remain powerful, but their application to hominin behaviour requires caution, recognition of behavioural contingency and plasticity and careful attention to the boundaries of what available data can substantiate. Only through such caution can we avoid overstating inferences about extinct ancestors and build more robust hypotheses about human evolutionary history.

## Data Availability

No formal data is available associated with this manuscript.
