# Supplementary material for: Parsimony, homology and the continuity of stone tool use in the hominin clade
Source: Camb Prism Extinct. 2026 Aug 6;4:e11. doi: 10.1017/ext.2026.10018 (PMC13458300; doi:10.1017/ext.2026.10018)
Supplement: Peer review history

# Reviewer report: Review: Parsimony, homology and the continuity of stone tool use in the hominin clade — R0/PR2

**DOI:** 10.1017/ext.2026.10018.pr2

**Authors:** Metin Eren

**Affiliations:** Kent State University, United States

This reviewer is Metin I. Eren, waiving anonymity as is my usual practice. I found this manuscript to be an interesting, measured review, and I recommend that it can be published as is. While I have published on both the earliest stone technologies in the hominin lineage and on non-human primates, I would not consider either one of these topics my specialty. So, I may have missed some technical aspects (e.g., dates, fossils) of this manuscript that colleagues who do specialize in those areas can comment on better than me. But this manuscript correctly emphasizes the fact that while parsimony is a powerful inferential tool, parsimonious inferences are ultimately hypotheses to be tested with empirical data, not accepted facts. And if the data aren’t there or are unclear, then we should be honest about the inferential nature of our conclusions, which could mean they are incorrect. I not only believe that this manuscript will be read and cited, but I could see myself assigning it to my students to read for class.

# Reviewer report: Review: Parsimony, homology and the continuity of stone tool use in the hominin clade — R0/PR3

**DOI:** 10.1017/ext.2026.10018.pr3

**Authors:** Adela Cebeiro Munin

**Affiliations:** New York University, United States

Reviewer comments to the author(s)

This is Adela Cebeiro (I am happy to waive my anonymity). This paper presents an alternative perspective on the origins of stone tool use in hominin evolution, suggesting that convergent evolution is a stronger hypothesis than homology, based on a detailed analysis of behavioral, fossil, and archaeological data. From the onset, a clear strength of this paper is to highlight the need to think about stone tool use in the LCA of *Homo*-*Pan* not as a resolved issue, but as an open question that needs to be empirically tested. Moreover, the authors make very good points across the manuscript: (1) the idea that early stone tool use emerges through independent innovations in different extant taxa, and how this could be applied to hominin evolution; (2) the fact that it is important to have a clear distinction between morphological and behavioral characteristics, and how that highlights the need to be cautious when it comes to using phylogenetic tools to reconstruct the evolution of behavioral and cultural traits; (3) how choosing chimpanzees instead of bonobos as the stone tool-using reference taxon could lead to infer that the LCA used stone tools; and (4) the notion that chimpanzees, bonobos, and humans have all experienced around 7 mya of evolution from the LCA, and how the fossil and archaeological evidence of *Pan* is too scarce to present a direct comparative scenario with the hominin records. Overall, this is a relevant paper that opens a perspective that has not been addressed as directly before. I recommend <b>the paper to be accepted</b> with some <b>minor revisions</b>.

<b><u>Minor revisions and suggested areas of improvement</u></b>

Although I believe the paper is well written and clearly exposes its relevance for the discipline, the following are some suggestions that could strengthen your argument.

● I am not sure the three lines of evidence you discuss in the abstract correspond to what you then mention in the main text of the manuscript. In the abstract, you refer to “(...) behavioral data from extant primates (...); the hominin fossil and archaeological record (...); and comparative data from phylogenetically distant tool-using animals” (lines 45-49). However, at the end of section 2, “Parsimony: interpreting phylogenetic relationships based on cladistics,” you mention the following complementary areas: “1) the variation in tool-use across extant stone tool using primates, 2) anatomical capacity for tool-use exhibited in the fossil hominin record and 3) the discontinuous and temporally delayed emergence of stone tool technology evident in the archaeological record” (lines 189-192). I also think the third point raised in the abstract (“comparative data from phylogenetically distant tool-using animals”) is somewhat similar to the first one. Overall, I would adjust the abstract to refer to the same three areas you discuss in section 2, with a focus on extant tool-using primates, the fossil record, and the archaeological record.

○ In relation to this, I think you could merge section 3, "Evidence from *Pan*,” with section 5, “Evidence from other extant animal species.” Although I understand why you separated them, I think it could be a good idea to combine them since they are both quite related. You could then expand on the fossil and archaeological record section and have them as two separate ones to match what you said at the end of section 2.

● I do not think this is an essential change, but I would advise caution referring to a “clear fossil” record for early *Homo* (line 258) when it comes to evidence for tool-use, just because we have quite a scarce and controversial situation regarding hand bones for *Homo*, particularly between 2.6-2.9 mya that you refer to since we do not really have hand bones for that period (to my knowledge). For example, there are only three complete hominin hands before Neanderthals: StW 573 or “Little Foot,” *Australopithecus sediba* MH2, and *Homo naledi* Hand 1 (Berger et al. 2015; Clarke 1999; Kivell et al. 2011, 2015, 2018; see Kivell 2015; Kivell et al. 2023 for an overview).

● Following up from my previous comment, I think you should adjust the paragraph discussing the anatomical capacity for tool use in association with hand grips (lines 278-286). From my perspective, multiple things could be problematic:

○ The “precision pad-to-side” grip type is actually quite common in extant primates (e.g., Bardo et al. 2017; Marzke et al. 2015; Marzke and Wullstein 1996; Westergaard and Suomi 1997), which in a strict sense would not make it an essential characteristic to produce Oldowan, and even less so the Acheulean. Moreover, even though it is true that having a long thumb and broad distal phalanges facilitates the use of certain precision grips, particularly pad-to-pad, this does not mean that those anatomical characteristics are essential for precision grips, and even less so for pad-to-side. For example, some recent studies show that *Pan* could engage in somewhat forceful pad-to-side grips even if not to the same degree as humans (e.g., Cebeiro and Key 2023; Gumert et al. 2009<sup>1</sup>; Marzke et al. 2015; Neufuss et al. 2017), and even if not forceful, they can definitely do pad-to-side grips.

○ I think talking about “three-jaw chuck grips” is too vague. There are many different variations of those types of grips, and they are not all directly associated with the Oldowan and the Acheulean. For example, Marzke and Wullstein (1996) classify the following grips as three-jaw chuck when comparing chimps with humans: tip-to-tip, distal finger pad-to-pad, extended three-jaw chuck, amongst others. Another more recent example is Bardo et al. (2016), which describes the following as a three-jaw chuck when studying bonobos during tool use tasks: three-jaw chuck, and three-jaw chuck with thumb. Here, if you want to refer to a three-jaw chuck grip that is normally associated with stone tool use, you should refer to the “three-jaw pad-to-pad” (e.g., Key et al. 2018) to avoid any confusion. Overall, the hand grip literature is a bit confusing and unclear.

○ Another interesting grip you could talk about that is normally associated with stone tool making is the “cradle grip,” since it has only been seen in humans (e.g., Key et al. 2019).

○ If we were specific, the anatomical features you mention are a bit controversial when it comes to saying that they are present in “all known *Homo* lineages” since we do not really know due to the scarcity of hand bones in the fossil record (see above).

○ I would add some more references in this section, expanding from Marzke to some of her more recent papers, as well as other recent papers by other authors. For example, some of the main references from the articles I have mentioned so far would be Neufuss and colleagues (2017) and Key and colleagues (2018).

○ If you want to be specific in the types of grips that are currently associated with stone tool use and making, it is best to refer to forceful precision grips (e.g., Marzke 2013). Even then, there is a need to be cautious because it is possible that some extant primates are capable of forceful pad-to-side grips, as I mentioned above. The key distinction here is forceful pad-to-pad grips, which seem that no other primate can really do (although I am not totally sold on this).

● In the paragraph talking about robust capuchin monkeys and their value as comparative cases of habitual stone tool-use (lines 407-428), I would also mention *Sapajus xanthosternos* (Canale et al. 2009; Proffitt et al. 2025 [which you include but I would make it more explicit]) and *Sapajus flavius* (Lima et al. 2024).

● I think you should include the reference Goldsborough et al. (2024) at the end of this sentence: “A study of this behavior found that stone tool-use is practiced primarily by males, and that different levels of stone tool-use is observed across the islands, despite the presence of adequate stone and encased food resources throughout” (lines 435-438).

Finally, I would like to highlight some minor edits in the text regarding some typos and missing italics across some genera and species names.

● Lines 201-209: I am not sure whether I am missing something regarding species and subspecies conventions, but I believe you refer to chimpanzee “subspecies” across the text, but then you use the species names. For example, *Pan troglodytes* (lines 203, 204-205), *Pan schweinfurthii* (line 205), *Pan versus* (line 205), and *Pan eliotti* (line 206). In these cases, I believe the subspecies nomenclature is Pan troglodytes troglodytes, *Pan troglodytes schweinfurthii*, *Pan troglodytes verus* (note it is *verus* and not “versus”), and *Pan troglodytes eliotti*. Particularly, you use the word subspecies in the following lines: 202, 204, 206, and 210. Interestingly, you refer to them as “species” in line 215.

● Lines 208-209: the first parentheses by “(although see…” is not closed. You could close it after “...in Cameroon) (Morgan and Abwe 2006),” or include the reference within the parentheses, “...in Cameroon; Morgan and Abwe 2006).”

● Line 264: in “(...) *H helmei, H rhodesiensis*” they need to be *H. helmei* and *H. rhodesiensis* with the periods next to the H.

● There are multiple *Homo* across the article that need to be in italics. Here are all of the text lines where this happens: 267, 269, 272, 276, 278, 283, 289, 309, and 521.

● Line 293: there should be a period at the end of the sentence after (Leakey 1971).

● The need to include italics also happened with *Paranthropus* in lines 304, 310, and 313.

● Line 307: I think there is a typo when you wrote “(...) requiring tool -se at all.” Did you want to say “requiring a tool set at all”?

● Line 313: there should be a period after the P in *P. boisei*. Moreover, *boisei* needs to be in lowercase.

● Line 362: *ramidus* needs to be in lowercase.

● Line 377: *platyops* needs to be in lowercase.

● Lines 380 and 382: I believe here australopith should not be italicized since this is not the scientific genus name, but I might be missing something.

● Line 419: there should be either a space between “(e.g., social display)(Falótico and Ottoni 2016),” or include the reference within the parentheses, “(e.g., social display; Falótico and Ottoni 2016).”

● Line 423: “multifaceted” is in a different font.

● Line 430: here you should say *C. capucinus imitator* to keep consistent with the first reference to the subspecies in lines 409-410, and if you want to consider them a subspecies of the Atlantic white-faced capuchin, like they chose to do in Barrett and colleagues’ (2018) paper.

● Lines 448-449: I believe here *Macaca* is missing from the “(...) the three taxa that use stone tools most in the wild (*Pan, and Sapajus*).” I would also consider adding *Cebus* given the nut-cracking evidence in white-faced capuchins.

● I believe it will be better to stick to the convention of abbreviating the genus name across species after mentioning the species for the first time. I have noticed you do that sometimes, but not always. For example, you could abbreviate the genus name in *Kenyanthropus platyops* (line 520).

<sup>1</sup> Although Gumert and colleagues (2009) refer to precision grips in macaques, they do not specify which type. However, I believe it is safe to assume it will be at least pad-to-side.

<b><u>References</u></b>

●Bardo A, Borel A, Meunier H, et al (2016) Behavioral and functional strategies during tool use tasks in bonobos. Am J Phys Anthropol 161:125–140. https://doi.org/10.1002/ajpa.23015

●Bardo A, Cornette R, Borel A, Pouydebat E (2017) Manual function and performance in humans, gorillas, and orangutans during the same tool use task. Am J Phys Anthropol 164:821–836. https://doi.org/10.1002/ajpa.23323

●Berger LR, Hawks J, De Ruiter DJ, et al (2015) Homo naledi, a new species of the genus Homo from the Dinaledi Chamber, South Africa. eLife 4:e09560. https://doi.org/10.7554/eLife.09560

●Canale GR, Guidorizzi CE, Kierulff MCM, Gatto CAFR (2009) First record of tool use by wild populations of the yellow‐breasted capuchin monkey (Cebus xanthosternos) and new records for the bearded capuchin (Cebus libidinosus). Am J Primatol 71:366–372. https://doi.org/10.1002/ajp.20648

●Cebeiro A, Key A (2023) Captive bonobos (Pan paniscus) apply precision grips when using flaked stone tools. Am J Biol Anthropol 183:e24759. https://doi.org/10.1002/ajpa.24759

●Clarke RJ (1999) Clarke RJ. 1999 Discovery of complete arm and hand of the 3.3 million-year-old Australopithecus skeleton from Sterkfontein. S. Afr. J. Sci. 95, 477 – 480. South Afr J Sci 477–480

●Goldsborough Z, Crofoot MC, Barrett BJ (2024) Male‐biased stone tool use by wild white‐faced capuchins ( Cebus capucinus imitator ). Am J Primatol 86:e23594. https://doi.org/10.1002/ajp.23594

●Gumert MD, Kluck M, Malaivijitnond S (2009) The physical characteristics and usage patterns of stone axe and pounding hammers used by long‐tailed macaques in the Andaman Sea region of Thailand. Am J Primatol 71:594–608. https://doi.org/10.1002/ajp.20694

●Key A, Dunmore CJ, Marzke MW (2019) The unexpected importance of the fifth digit during stone tool production. Sci Rep 9:16724. https://doi.org/10.1038/s41598-019-53332-w

●Key A, Merritt SR, Kivell TL (2018) Hand grip diversity and frequency during the use of Lower Palaeolithic stone cutting-tools. J Hum Evol 125:137–158. https://doi.org/10.1016/j.jhevol.2018.08.006

●Kivell TL (2015) Evidence in hand: recent discoveries and the early evolution of human manual manipulation. Philos Trans R Soc B Biol Sci 370:20150105. https://doi.org/10.1098/rstb.2015.0105

●Kivell TL, Baraki N, Lockwood V, et al (2023) Form, function and evolution of the human hand. Am J Biol Anthropol 181:6–57. https://doi.org/10.1002/ajpa.24667

●Kivell TL, Churchill SE, Kibii JM, et al (2018) The hand of Australopithecus sediba. PaleoAnthropology 282–333

●Kivell TL, Deane AS, Tocheri MW, et al (2015) The hand of Homo naledi. Nat Commun 6:8431. https://doi.org/10.1038/ncomms9431

●Kivell TL, Kibii JM, Churchill SE, et al (2011) Australopithecus sediba hand demonstrates mosaic evolution of locomotor and manipulative abilities. Science 333:1411–1417. https://doi.org/10.1126/science.1202625

●Lima GCB, Lacerda JC, Taynor R, et al (2024) A new addition to the toolbox: stone tool use in blonde capuchin monkeys (Sapajus flavius). Primates 65:383–389. https://doi.org/10.1007/s10329-024-01143-7

●Marzke MW (2013) Tool making, hand morphology and fossil hominins. Philos Trans R Soc B Biol Sci 368:20120414. https://doi.org/10.1098/rstb.2012.0414

●Marzke MW, Marchant LF, McGrew WC, Reece SP (2015) Grips and hand movements of chimpanzees during feeding in Mahale Mountains National Park, Tanzania. Am J Phys Anthropol 156:317–326. https://doi.org/10.1002/ajpa.22651

●Marzke MW, Wullstein KL (1996) Chimpanzee and human grips: A new classification with a focus on evolutionary morphology. Int J Primatol 17:117–139. https://doi.org/10.1007/BF02696162

●Neufuss J, Humle T, Cremaschi A, Kivell TL (2017) Nut‐cracking behaviour in wild‐born, rehabilitated bonobos (Pan paniscus): a comprehensive study of hand‐preference, hand grips and efficiency. Am J Primatol 79:e22589. https://doi.org/10.1002/ajp.22589

●Proffitt T, De Sousa Medeiros P, Martins WP, Luncz LydiaV (2025) Flake production: A universal by-product of primate stone percussion. Proc Natl Acad Sci 122:e2420067122. https://doi.org/10.1073/pnas.2420067122

●Westergaard GC, Suomi SJ (1997) Capuchin monkey (Cebus apella) grips for the use of stone tools. Am J Phys Anthropol 103:131–135. https://doi.org/10.1002/(SICI)1096-8644(199705)103:1%3C131::AID-AJPA9%3E3.0.CO;2-Z

# Editor report: Recommendation: Parsimony, homology and the continuity of stone tool use in the hominin clade — R0/PR4

**DOI:** 10.1017/ext.2026.10018.pr4

**Affiliations:** University of Cambridge, United Kingdom of Great Britain and Northern Ireland

The authors provide a well written research article addressing the question of whether or not the LCA of Pan and Homo manufactured and used stone tools, and if there was a continuous record of stone tool use through the Miocene-to-Pleistocene. I agree with both reviewers that the manuscript is near publishable, but would recommend that the authors carefully consider the detailed and helpful comments of R2 during the revision process. Please note that an impact statement is required for the journal format, as is a data availability statement. As part of the revision process, it would be appreciated if the authors could keep in mind the length of the article, or even reduce its current length if possible.

# Editor report: Decision: Parsimony, homology and the continuity of stone tool use in the hominin clade — R0/PR5

**DOI:** 10.1017/ext.2026.10018.pr5

**Affiliations:** School of Natural Sciences, Macquarie University, Australia

No accompanying comment.

# Editor report: Recommendation: Parsimony, homology and the continuity of stone tool use in the hominin clade — R1/PR7

**DOI:** 10.1017/ext.2026.10018.pr7

**Affiliations:** University of Cambridge, United Kingdom of Great Britain and Northern Ireland

Thanks are extended to the authors for their revised manuscript. I am happy to recommend the article be accepted, but note the below very minor changes. Potentially they could all be made at the proofing stage, but to be on the safe side I am returning the manuscript back to the authors to address these very minor points. 

Line 88-89: Check the phrasing of ‘…relative to working competing hypotheses…’

Line 91: ‘mimimize’. British spelling is used elsewhere. 

Line 98: ‘…straightforward for anatomical morphological…’. Addition of ‘anatomical’ required for clarity. 

Line 162: ‘Prioritizing’. British spelling used elsewhere. 

Line 243: ‘epitomize’. British spelling used elsewhere. 

Line 274: Neither of these references actually evidence a continuous archaeological record (See Key and Williams, this special issue). 

Line 313: Paranthropus needs to be in italics. 

Line 315: ‘specialized’. British spelling used elsewhere. 

Line 318: Homo needs to be in italics. 

Line 364: ‘…broad distal phalanges’. Do the authors mean the apical tuffs?

Line 366: A citation to Kivell et al. (2023) is needed to support this statement (see R2’s prior comments). Currently there is no evidence to support this statement of fact.

Line 369: ‘The fossil evidence support…’. Check phrasing. 

Line 379: ‘…user, however, and the…’. Addition of ‘and’ may be needed. 

Line 390: ‘The ESA archaeological record…’. Addition of ‘ESA’ may be needed to clarity. 

Line 424: ‘specialized’. British spelling used elsewhere. 

Line 470: ‘…gap between the LCA (6-7 Ma) and the earliest…’. Addition of two ‘the’ may be needed. 

# Editor report: Decision: Parsimony, homology and the continuity of stone tool use in the hominin clade — R1/PR8

**DOI:** 10.1017/ext.2026.10018.pr8

**Affiliations:** School of Natural Sciences, Macquarie University, Australia

No accompanying comment.

# Editor report: Recommendation: Parsimony, homology and the continuity of stone tool use in the hominin clade — R2/PR10

**DOI:** 10.1017/ext.2026.10018.pr10

**Affiliations:** University of Cambridge, United Kingdom of Great Britain and Northern Ireland

Many thanks for making these last few minor additions. I am now happy to recommend acceptance.

# Editor report: Decision: Parsimony, homology and the continuity of stone tool use in the hominin clade — R2/PR11

**DOI:** 10.1017/ext.2026.10018.pr11

**Affiliations:** School of Natural Sciences, Macquarie University, Australia

No accompanying comment.
